# Accuracy and reliability analysis of a machine learning based segmentation tool for intertrochanteric femoral fracture CT

**DOI:** 10.3389/fsurg.2022.913385

**Published:** 2022-07-26

**Authors:** Dongdong Wang, Zhenhua Wu, Guoxin Fan, Huaqing Liu, Xiang Liao, Yanxi Chen, Hailong Zhang

**Affiliations:** ^1^ Department of Orthopaedics, Shanghai General Hospital, Shanghai Jiaotong University School of Medicine, Shanghai, China; ^2^ Department of Orthopaedic Trauma, Shanghai East Hospital, Tongji University School of Medicine, Shanghai, China; ^3^ Sun Yat-Sen University School of Computer Science and Engineering, Shenzhen, China; ^4^ Department of Pain Medicine, Huazhong University of Science and Technology Union Shenzhen Hospital, Shenzhen, China; ^5^ Guangdong Key Laboratory for Biomedical Measurements and Ultrasound Imaging, School of Biomedical Engineering, Shenzhen University Health Science Center, Shenzhen, China; ^6^ Department of Spine Surgery, Third Affiliated Hospital, Sun Yat-Sen University, Guangzhou, China; ^7^ Artificial Intelligence Innovation Center, Research Institute of Tsinghua, Guangzhou, China; ^8^ Department of Orthopaedic Surgery, Zhongshan Hospital, Fudan University, Shanghai, China; ^9^ Department of Orthopaedics, Putuo People’s Hospital, Tongji University, Shanghai, China

**Keywords:** machine learning, intertrochanteric femoral fracture, semantic segmentation, 3D reconstruction, computed tomography

## Abstract

**Introduction:**

Three-dimensional (3D) reconstruction of fracture fragments on hip Computed tomography (CT) may benefit the injury detail evaluation and preoperative planning of the intertrochanteric femoral fracture (IFF). Manually segmentation of bony structures was tedious and time-consuming. The purpose of this study was to propose an artificial intelligence (AI) segmentation tool to achieve semantic segmentation and precise reconstruction of fracture fragments of IFF on hip CTs.

**Materials and Methods:**

A total of 50 labeled CT cases were manually segmented with Slicer 4.11.0. The ratio of training, validation and testing of the 50 labeled dataset was 33:10:7. A simplified V-Net architecture was adopted to build the AI tool named as IFFCT for automatic segmentation of fracture fragments. The Dice score, precision and sensitivity were computed to assess the segmentation performance of IFFCT. The 2D masks of 80 unlabeled CTs segmented by AI tool and human was further assessed to validate the segmentation accuracy. The femoral head diameter (FHD) was measured on 3D models to validate the reliability of 3D reconstruction.

**Results:**

The average Dice score of IFFCT in the local test dataset for “proximal femur”, “fragment” and “distal femur” were 91.62%, 80.42% and 87.05%, respectively. IFFCT showed similar segmentation performance in cross-dataset, and was comparable to that of human expert in human-computer competition with significantly reduced segmentation time (*p *< 0.01). Significant differences were observed between 2D masks generated from semantic segmentation and conventional threshold-based segmentation (*p *< 0.01). The average FHD in the automatic segmentation group was 47.5 ± 4.1 mm (41.29∼56.59 mm), and the average FHD in the manual segmentation group was 45.9 ± 6.1 mm (40.34∼64.93 mm). The mean absolute error of FHDs in the two groups were 3.38 mm and 3.52 mm, respectively. No significant differences of FHD measurements were observed between the two groups (*p *> 0.05). All ICCs were greater than 0.8.

**Conclusion:**

The proposed AI segmentation tool could effectively segment the bony structures from IFF CTs with comparable performance of human experts. The 2D masks and 3D models generated from automatic segmentation were effective and reliable, which could benefit the injury detail evaluation and preoperative planning of IFFs.

## Introduction

Intertrochanteric femoral fracture (IFF) is commonly seen in the elderly women with severe osteoporosis (1), and is increasingly prevalent as the population continues to age (2). Concurrently, surgical interventions especially internal fixation is the most common treatment for stable and unstable fractures due to the advantage of early rehabilitation and mobilization (3). The surgical goals are to achieve stable fixation, allow early mobilization and improve the quality of life of patients (1, 4). To assist the surgery, computed tomography (CT) and three-dimensional (3D) CT are widely used in the clinical settings for precise evaluation and diagnosis (4). Furthermore, CT and 3D reconstruction are considered as pre-requisites for computer-assisted preoperative planning, intraoperative navigation and postoperative assessment of managing IFFs and other hip diseases (5–9). The fracture details on CT with 3D reconstruction can assist orthopaedic surgeons to achieve better understanding of the morphologic characteristics and injury mechanisms. As a result, the surgeons would benefit from planning the optimal surgical approaches, achieving anatomical fracture reduction and decreasing fixation failures.

However, reliable 3D reconstruction depends on the accurate segmentation of target tissues on CT images. Manual 3D segmentation is labor intensive and time-consuming, for that typical CT volumes of one patient usually contain hundreds of 2D image slices (10). Besides, the segmentation accuracy also highly depends on the technicians who conduct the segmentation (11). Thus, automatic segmentation based on machine learning methods is proposed and used in many studies (9–17). These machine learning methods are usually divided into two main categories: supervised learning and unsupervised learning, which depends upon whether prior knowledge was utilized or not. Unsupervised methods including thresholding (18), region growing (19), graph cut (20) and so on were applied. Accordingly, these segmentation models did demonstrate the effectiveness and accuracy in hip segmentation but were suitable only for solving certain hip segmentation under healthy or minimal pathological conditions. Supervised methods including statistical shape model (15, 21), atlas-based (14) and deep learning (10, 17) methods were also applied, but they were usually established with large amount of training data to achieve satisfactory segmentation result, which was also time-consuming and tedious. However, the automatic segmentation remains a challenging task due to the characteristic features of hip CTs, which includes the inherent blur of CT images, the weak boundaries between pelvis and femur, the narrowness of joint space, the low quality of CT scans and the patient’s leg posture (9, 10). Furthermore, these studies usually focused on the femur segmentation from hip CTs, but not the semantic segmentation of fracture fragments.

To the best of our knowledge, no studies were available on achieving automatic segmentation and precise reconstruction of fracture fragments (including head-neck, femoral shaft, lesser trochanter, greater trochanter, lateral wall, posterior crest, anterior cortex fragment and so on) on hip CTs, which could be helpful for fracture evaluation and surgical design of IFFs. Thus, the purpose of the present study was to propose an artificial intelligence (AI) segmentation tool to achieve semantic segmentation and precise reconstruction of fragments of IFFs on hip CTs.

## Materials and methods

**Figure 1:**
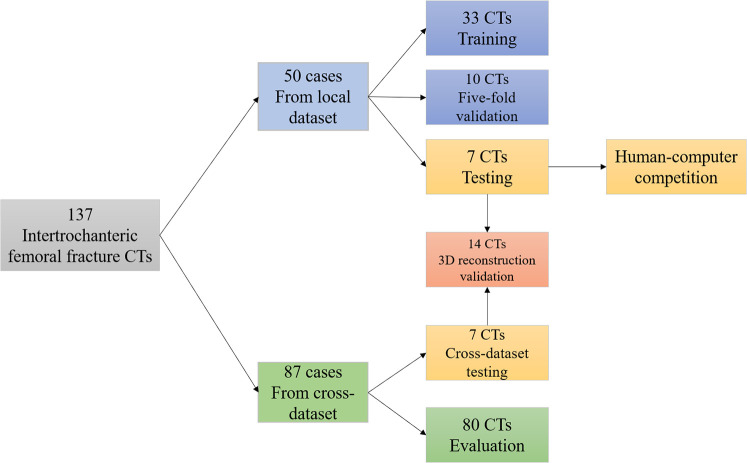
Data sources and distribution of 137 intertrochanteric femoral fracture CTs.

This retrospective study was Health Insurance Portability and Accountability Act compliant and was approved by the institutional ethical committee of local institutions before data extraction. The medical records and imaging data of IFF patients in our institutions were retrospectively obtained and reviewed. The inclusion criteria were: (1) IFF patients confirmed by medical records and radiological images; (2) with thin-layer CTs; (3) with 1 or more fracture fragments on CT. The exclusion criteria were: (1) metal artifacts on CT; (2) combined with pathological fractures, hip tumor, hip deformity, or other combined fractures. A total of 137 patients with thin-layer hip CTs were included in this study, in which 50 labeled CTs from institution A was regarded as the local dataset for the development of the AI segmentation tool, and the other 87 CTs (7 labeled and 80 unlabeled) from institution B was regarded as the cross-dataset for testing and segmentation evaluation (Figure 1).

All image annotations were conducted in a personal computer (graphics processing unit: a Nvidia GeForce 1080Ti, with 4 GB of memory and a 3.5-GHz Intel Core i7–4790 CPU with 8 GB of memory). The machine learning model for semantic segmentation was developed, trained, validated and tested using Keras (version 2.1.1 with tensorflow_backend py).

### Manual annotation

**Figure 2:**
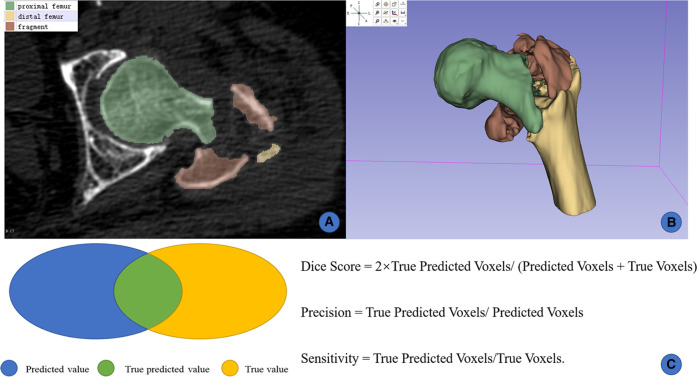
Manual segmentation and 3D reconstruction on slicer. (**A**) manual labels by human experts; (**B**) 3D reconstruction from 2D masks; (**C**) illustration of the Dice score, precision, and sensitivity.

A total of 50 CT cases were manually segmented with Slicer 4.11.0 (http://www.slicer.org). The bony structures of the proximal femur on hip CTs were meticulously segmented and labeled. An orthopaedic surgeon who was an expert in reading hip CT and had systematic training in Slicer manually was required to segment the bones manually. According to the location and clinical concepts of different fragments, the bony structures were simply divided into three categories: the “proximal femur”, the “fragment” and the “distal femur” (Figure 2). Then the segmented images were reviewed by a radiologist expert and another orthopaedic surgeon, both of whom were experienced in hip CT reading. Any disagreement of segmentation was solved by the vote of three doctors. These manual annotations were regarded as the ground truth.

### Data preprocessing and segmentation assessment

All thin-layer CTs were preprocessed using the following steps: resampling, cropping, and intensity normalization. The Dice score, precision and sensitivity were used to assess the segmentation performance of all fragments (Figure 2). The functions of these 3 indicators were as follows:

Dice Score = 2 × True Predicted Voxels/(Predicted Voxels + True Voxels)
Precision = True Predicted Voxels/Predicted Voxels
Sensitivity = True Predicted Voxels/True Voxels

Because the Dice score was the most common indicator in computer vision, the current study adopted it as the primary index to assess the segmentation performance of the AI tool.

### Network architecture

**Figure 3:**
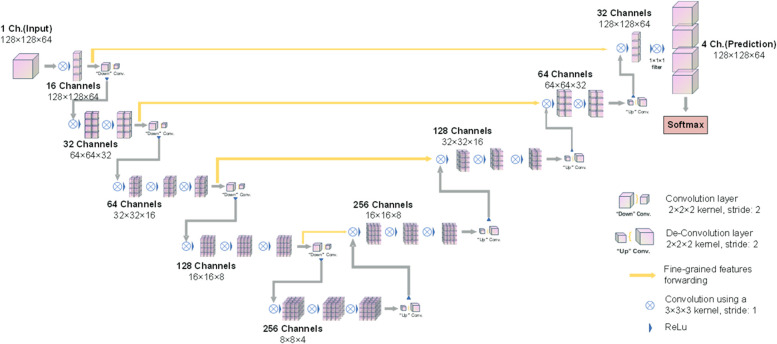
Schematic of the simplified V-Net network architecture (IFFCT).

The presented AI segmentation tool was developed based on the V-Net architecture (22). The 5-layer simplified V-Net architecture consists of two parts, including the encoder and the decoder parts (Figure 3). The encoder part performed data analysis and feature-representation learning from the input data, and the decoder part generates segmentation results. The Rectified Linear Unit (ReLU) nonlinear activation function was used in the whole network structure. There were also 4 shortcut connections (concatenations) between layers of equal resolution in the encoder and decoder paths. The last layer of the model was a 1 × 1 × 1 convolutional layer followed by a softmax layer, with 4 output channels. The input of the model was 128 × 128 × 64 voxel patches of CT, the output was the corresponding probability mask with the shape of 128 × 128 × 64 × 4. The developed AI segmentation tool was named as **IFFCT** (Intertrochanteric Femoral Fracture CT) because its aim was to automatically segment multiple bony fragment structures of IFF CTs.

### Training

During the training phase, the original catenary data was subjected to the standard processing and the augmentation operation in each training iteration, including add random scaling, Gaussian noise, rotation, flipping and cropping. The augmented training data were used as the input, this could alleviate the risk of insufficient generalization performance caused by the small amount of training data. Dice coefficient Loss Function was used instead of Cross-Entropy Loss Function as the loss function, which was one of the characteristics of V-Net. The convolutional layer parameters of V-Net were initialized by the method of He et al (23). The size of the patch (depth × height × width) input to the V-Net neural network during the training process was 128 × 128 × 64 (unit: voxel); the minibatch was 8, optimized by the SGD-M optimization algorithm; and the initial learning rate was 1e^−2^. The reason we chose the SGD-M optimization algorithm instead of Adam optimization algorithm for training was its stable performance, not easy to fall into the local optimum, and high computational efficiency.

### Five-fold validation

During the training process, for each patch *x_i_* in the original CT image *X*, model *M* would output the corresponding probability mask *y_i_*, *y_i_* = *M*(*x_i_*). We used the combined algorithm (Table 1) to sum the label probabilities of every patch according to its locations. Then, the automatic segmented mask was obtained. By comparing the automatic segmented masks with the manual segmented masks, the Dice scores of various kinds of voxel classes could be obtained. Five-fold validation was conducted using the validation dataset in order to select the final model IFFCT with the highest segmentation performance.

**Table 1:** Overview of the combined algorithm.

Algorithm 1: Combined Algorithm
**Require:** X: CT volume, H × W × D **Require:** *x_i_*∈ *X(L_i_),* (*i* = *1,……, k*): CT voxel patch **Require:** *y_i _*= *M*(*x_i_*): *y_i_*is the output of the last layer (SoftMax activation function) of the model M, *y_i_* has 1 more dimension than *x_i_*, and this dimension has 4 channels. Each channel refers to the probability of the corresponding voxel belonging to background, proximal femur, fragment or distal femur, respectively.
(1) **For** *x_i_*∈ *X(L_i_),* (*i* = *1,……, k*) **do** (2) *Y*(*L_i_*) + *y_i_* (3) *N(L_i_)* + 1 (4) **End for** (5) *S* ← arg max (*Y/N*, axis = −1) (find the channel with the largest value in the last dimension) (6) **Return** *S* (the automatic mask)

### Testing

The ratio of training, validation and testing of the 50 labeled cases was 33:10:7. A total of 7 cases randomly selected from the local dataset were tested on IFFCT. Another 7 cases from cross-dataset (IFF CTs from another institution) were also tested in order to validate the robustness of the trained model. To obtain the ground truth of these CTs from cross-dataset, the same surgeons and radiologist conducted the annotation and review. The Dice score, precision and sensitivity were used to assess the segmentation performance of all bony structures of IFF CTs (Figure 2).

In order to compare the segmentation performance of IFFCT with human experts, we also introduced the human-computer competition. Namely, the 7 cases from the local test dataset were segmented by another orthopaedic expert who had CT reading and Slicer operation experiences. Then, the Dice scores of manual segmentation were calculated by comparing with the ground truth. Segmentation time of local test dataset and cross-dataset of by IFFCT or human experts were also recorded.

### Evaluation of AI-generated masks by human experts

**Figure 4:**
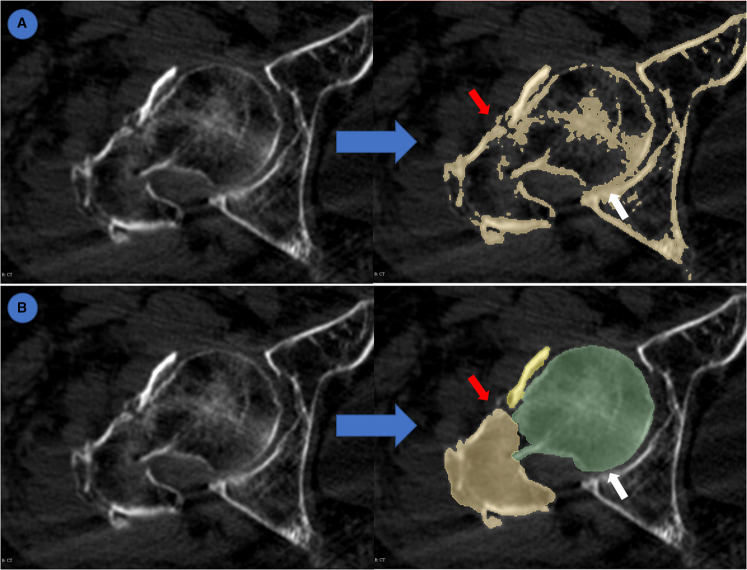
Evaluation of semantic segmentation and threshold-based segmentation. (**A**) threshold-based segmentation; (**B**) semantic segmentation. Blue arrow: from original CT to segmented masks. Red arrow: different fracture fragments were highlighted by different colors which made it easy to distinguish. White arrow: adhesion between pelvis and femur.

Given that the evaluation indexes are all indicators in computer vision, and may not represent the applicability in clinical practice, we further evaluated the segmentation accuracy of AI-generated masks and normal segmented masks. A total of 80 unlabeled CTs from cross-dataset were segmented using the normal threshold method and IFFCT-based semantic segmentation method on Slicer. Three independent observers reviewed and evaluated the segmented 2D masks according to the difficulties to distinguish each fracture fragment and the adhesion of the pelvis and femur (Figure 4). Any disagreement of segmentation evaluation was solved by the vote of three observers.

### 3D Reconstruction and measurement validation

**Figure 5:**
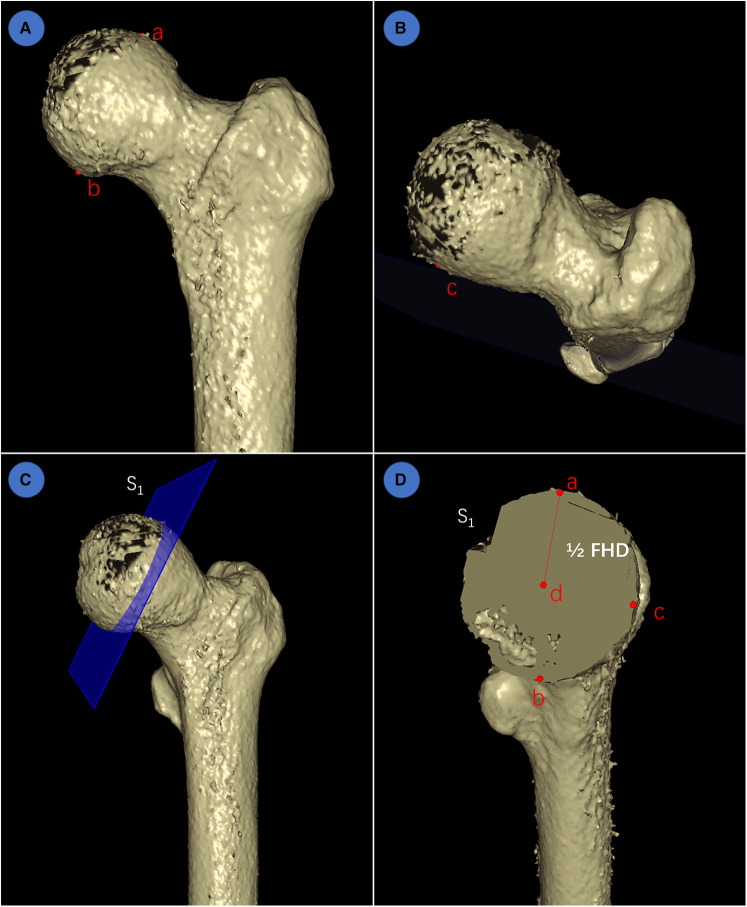
Schematic measurement of FHD in 3D space. (**A**) determination of the highest point a and the lowest point b at the maximum expanding region of femoral head at the anterior-posterior view; (**B**) determination of point c at the maximum expanding region of femoral head at the overlook view; (**C**) according to geometry, these three points could determine a plane S_1_; (**D**) the center point d, radius (line segment ad = 1/2 FHD) and diameter (FHD) could also be determined using geometry principle.

To further investigate the segmentation performance and reconstruction precision of IFFCT, we measured the femoral head diameter (FHD) on 3D models generated from both manually segmented and automatically segmented images. The schematic measurement of FHD in 3D space were listed below (Figure 5): first, the highest point a and the lowest point b at the maximum expanding region of the femoral head were selected at the anterior-posterior view. Then, point c at the maximum expanding region of the femoral head was also selected at the overlook view. According to the concepts of geometry, these three points could define a plane S_1_, and the diameter of the circumcircle of these three points could be defined as the FHD.

**Figure 6:**
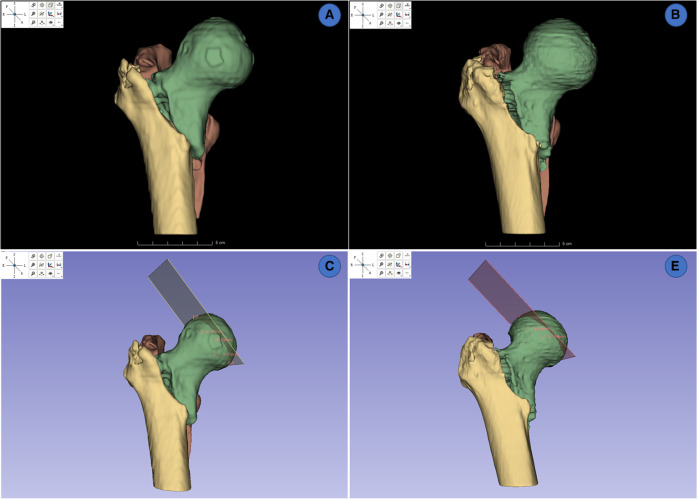
3d reconstruction and measurements of FHDs on slicer. (**A,B**) 3D rendering from manually segmented masks and automatically segmented masks. (**C,D**) measurement of FHD on 3D model from manually segmented masks and automatically segmented masks.

A total of 28 reconstructed models of IFF CT were then reconstructed and visualized on Slicer (Figure 6), 14 models were from the local test dataset and 14 models were from the cross-dataset. The ground truths of the manual segmentation in this part were identified by the experts mentioned above. Two independent observers measured the FHD of 3D models from manually and automatically segmented images. One month later, one of the observers measured the FHD again. The intraclass correlation coefficient (ICC) was calculated to assess test-retest reliability and inter-observer reliability of multiple measurements.

### Statistical analysis

All data were statistically analyzed by SPSS 22.0 (IBM Corporation, Chicago, USA). The normal distribution was tested using the Shapiro–Wilk test. Mann–Whitney U test was used to compare the differences of segmentation time between IFFCT and human experts. The difference of segmentation performance on 2D masks between IFFCT-based semantic segmentation and threshold-based segmentation were compared using chi-square test. The differences of FHD measurement on 3D models between automatically generated masks and manually generated masks were compared using a paired Student *t* test. The reliabilities of the 3D measurements between manually and automatically segmented images was compared using intra-observer and inter-observer ICCs. All continuous data were presented as mean ± SD, and *p* < 0.05 was considered statistically significant.

## Results

**Figure 7:**
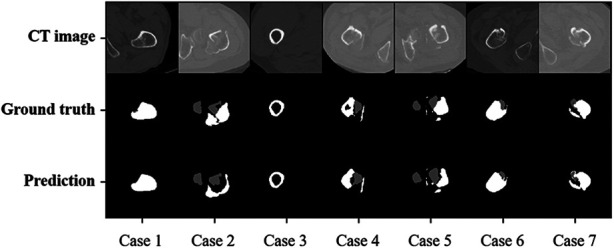
Automatic and manual labeled masks in local test dataset.

**Figure 8:**
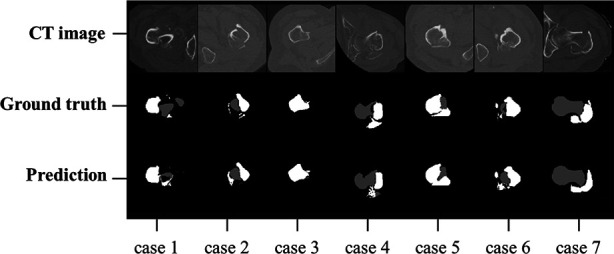
Automatic and manual labeled masks in cross-dataset.

Testing results showed that IFFCT could achieve successful segmentation of multiple bony structures encompassing “proximal femur”, “fragment” and “distal femur” on CT (Figure 7). The quantitative segmentation accuracy was shown in Table 2. The average Dice score, precision and sensitivity of “proximal femur” were 91.62%, 99.60% and 92.34%. The average Dice score, precision and sensitivity of “fragment” were 80.42%, 76.24% and 78.67%. In “distal femur”, the average Dice score, precision and sensitivity were 87.05%, 87.77% and 86.27%. The segmentation performance from cross-dataset was also shown in Table 3 and Figure 8. The average Dice score of the “proximal femur”, “fragment” and “distal femur” were 87.19%, 69.70% and 88.75%, respectively. This could reveal that our AI segmentation tool has the fair generalization ability.

**Table 2:** Segmentation accuracy in 7 testing cases from the local dataset.

Structures	Dice Score (%)	Precision (%)	Sensitivity (%)
Proximal femur	91.62	99.60	92.34
Fragment	80.42	76.24	78.67
Distal femur	87.05	87.77	86.27

**Table 3:** Segmentation accuracy in 7 testing cases from the cross-dataset.

Structures	Dice Score (%)	Precision (%)	Sensitivity (%)
Proximal femur	87.19	91.43	85.37
Fragment	69.70	74.02	76.47
Distal femur	88.75	91.50	86.55

**Figure 9:**
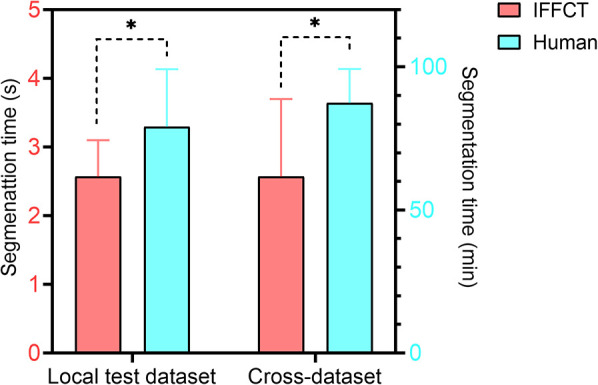
Segmentation time of human experts and IFFCT in local test dataset and cross-dataset. Local test dataset: 2.6 ± 0.5s vs 79.1 ± 20.1 min; cross-dataset, 2.6 ± 1.1s vs 87.4 ± 11.9 min. *P < 0.01.

After training, it took about 2.6 s for IFFCT to complete an automatic segmentation (from data preprocessing to semantic segmentation) on a single case in local test dataset (2.6 ± 0.5 s) and cross-dataset (2.6 ± 1.1 s), which was significantly less than that of manual segmentation (local test dataset 79.1 ± 20.1 min, cross-dataset 87.4 ± 11.9 min) (Figure 9). Besides, we also conducted the human-computer competition, and the segmentation performance of IFFCT was comparable to that of human expert (Table 4).

**Table 4:** Segmentation accuracy of human experts in the Human-computer competition.

Structures	Dice Score (%)	Precision (%)	Sensitivity (%)
Proximal femur	95.16	91.64	90.81
Fragment	87.64	85.32	82.40
Distal femur	90.70	88.88	83.18

The results of segmentation performance on 2D masks showed that there were significant differences between semantic segmentation and conventional threshold-based segmentation (Table 5). In the semantic segmentation group, 24 cases were evaluated as difficult to distinguish fracture fragments, while 57 cases were regarded as difficult ones in the threshold-based segmentation group (*p *< 0.01). There were 10 cases with the adhesion of pelvis and femur in the semantic segmentation group, while there were 26 cases in the threshold-based segmentation group (*p *< 0.01).

**Table 5:** The evaluation of segmentation accuracy of 2D masks generated from semantic segmentation and threshold-based segmentation.

	Semantic segmentation	Threshold-based segmentation	*P* value
Difficulty of distinguish fracture fragments			<0.01*
Difficult	24 (30.00%)	57 (71.25%)	
Easy	56 (70.00%)	23 (28.75%)	
Adhesion of pelvis and femur			<0.01*
With	10 (12.50%)	26 (32.50%)	
Without	70 (87.50%)	54 (67.50%)	

* *P* < 0.01, significant differences between the two groups.

The results of 3D measurement between 3D models generated from automatic segmentation and manual segmentation (ground truths from the local dataset and cross-dataset) were shown in Table 6. The average FHD in the automatic segmentation group was 47.5 ± 4.1 mm (41.29∼56.59 mm), and that of the manual segmentation group was 45.9 ± 6.1 mm (40.34∼64.93 mm). The mean absolute error (MAE) of FHD in the automatic segmentation group and manual segmentation group were 3.38 mm and 3.52 mm, respectively. No significant differences of FHD measurements were observed between the two groups (*p *> 0.05). The reliability test revealed strong test-retest reliability and inter-observer reliability in measurements on 3D models generated from automatic and manual segmentations (all ICC values >0.8).

**Table 6:** The measurement results of FHD (mm) and reliability assessments.

Parameters	Minimum	Maximum	Mean ± SD	MAE	Intra-observer ICC	Inter-observer ICC
Automatic segmentation	41.29	56.59	47.54 ± 4.10	3.38	0.874	0.856
Manually segmentation	40.34	64.93	45.90 ± 6.09	3.52	0.886	0.845

## Discussion

In this study, we constructed an AI segmentation tool based on the V-Net neural network for semantic segmentation of intertrochanteric fracture CTs and demonstrated the competency of the AI tool to segment fracture fragments when compared against qualified human experts. Results have showed a satisfying segmentation performance on the test dataset with the average Dice score over 80%. Meanwhile, the segmentation performance in the cross-dataset showed a satisfying generalization ability of our AI tool. The segmentation performance of our AI tool was not only roughly comparable to that of human experts, but also with a much less segmentation time. The segmentation evaluation on 2D masks revealed that the majority of the AI-generated masks were deemed satisfactory by the experts. The accurate semantic segmentation could facilitate the computer-assisted diagnosis, injury detail evaluation and preoperative planning procedures of IFF.

In addition, precise 3D reconstructions of relevant bony structures were also achieved with the assistance of our AI segmentation tool. No significant differences of FDH measurements were observed between automatically and manually generated 3D models. The 3D reconstruction with the assistance of our AI segmentation tool was efficient and reliable according to the results of test-retest reliability and inter-observer reliability of multiple measurements, in which all ICCs were greater than 0.8. Accordingly, recent studies have shown the advantages of CT reconstruction in fracture stability assessment and implants selection over X-rays (24, 25). When looking at the prominence of precise reconstruction, the 3D reconstruction of intertrochanteric fractures could assist surgeons observing fracture lines and fragments, better understanding the fracture mechanisms and evaluating the fracture stability in clinical settings. Surgeons could determine whether the operation and preoperative planning were needed or not, and it could also benefit the intraoperative fracture localization. Moreover, postoperative evaluation and fracture prognosis prediction could also benefit from the use of CT reconstructions.

Due to the defects of inefficiency, time-consuming and highly required experience of manual segmentation, many studies have focused on automatic segmentation based on machine learning methods. For example, Chen et al. (10) presented a 3D feature-enhanced network for hip segmentation with a high Dice similarity coefficient of 96.88% and average segmentation time of 0.93 s, but its performance heavily depends on large amount of training data, which were not always available. Chang et al. (17) have proposed a patch-based refinement algorithm for automatic femur segmentation from CT images. Their method achieved accurate segmentation on a small dataset of 60 CT hips (120 hemi-hips). In their study, the processing time was about 9s per CT volume. It should be noted that Chang’s method was more suited for segmentation of diseased hips when compared with Chen’s work. However, these femur segmentations were not semantic, and might not be directly used in the evaluation of fracture fragments on IFF CTs. Our study introduced an AI segmentation tool for automatic segmentation of the fracture fragments on IFF CTs through a simplified V-Net neural network and validated the feasibility and reliability of the 2D masks and 3D reconstructed models of relevant bony structures.

The presented AI tool in this study could be easily extended to other applications. One such extension might apply our AI tool to the segmentation of femoral head fracture, femoral neck fracture and subtrochanteric fracture. In the future, the AI tool will be trained on more datasets, especially on some CT of proximal femoral fracture patients and the necrosis of the femoral head. We hoped that the fracture fragments on other fracture CTs would also be accurately segmented and precisely reconstructed with the assistance of the AI tool. This would undoubtedly benefit the evaluation in clinical practice, enhance the understanding of morphologic fracture characteristics and injury mechanisms for orthopaedic surgeons, and facilitate surgical strategies as well as operation preparation.

There are several limitations of this study that should be noted. First, the segmentation performance of different fracture fragments was not consistent, the Dice score of “proximal femur” (91.62%) was higher than that of “fragment” (80.41%) and “distal femur” (87.05%). While in our IFFCT model, the lesser trochanter fragment, intermediate fragment and coronal fragment were included as the “fragment”. The relatively low segmentation accuracy of “fragment” may limit the application of our AI tool in fracture classification, for the new AO/OTA classification focuses on these fracture fragments and the femoral lateral wall (26). Next, deep learning methods for segmentation or other tasks usually need to leverage large amount of labeled data for model training (27). Although our AI tool has achieved satisfying segmentation accuracy on a small training dataset. These are the instinct characteristics of V-Net (or U-Net, 3D U-Net) that use data augmentation, residual learning or skip connections to learn from a small dataset. The presented AI segmentation tool was based on a simplified V-Net architecture, and residual function was not used. It might be the reason for not high enough segmentation accuracy. A semi-supervised approach for semantic segmentation by leveraging both limited labeled data and abundant unlabeled data would have the tremendous potential (28). We believe that the segmentation performance of our AI tool would be greatly improved with more training data, application of residual functions and semi-supervised learning methods in the future.

## Conclusion

In summary, we proposed an AI segmentation tool that could effectively segment the fracture fragments from IFF CTs with comparable performance of human experts. The 2D masks and 3D models generated from automatic segmentation are effective and reliable, which could benefit the injury detail evaluation and preoperative planning of IFFs without tedious and time-consuming segmentation and reconstruction procedures. Future studies with larger samples are needed to validate and improve the performance of our AI segmentation tool.

## Data Availability

The datasets presented in this study can be found in online repositories. The names of the repository/repositories and accession number(s) can be found below: The original contributions presented in the study are included in the article and supplementary material, and further inquiries (the local test dataset and cross dataset) can be directed to the corresponding author. The code is available at https://github.com/ArthurWuzh/unsegmented_prediction.git.
